# Predicting essential proteins by integrating orthology, gene expressions, and PPI networks

**DOI:** 10.1371/journal.pone.0195410

**Published:** 2018-04-10

**Authors:** Xue Zhang, Wangxin Xiao, Xihao Hu

**Affiliations:** 1 School of Medicine, Tufts University, Boston, MA, United States of America; 2 School of Computer and Software Engineering, Huaiyin Institute of Technology, Huai’an, Jiangsu, China; 3 Department of Biostatistics and Computational Biology, Dana-Farber Cancer Institute, Boston, MA, United States; 4 Harvard T.H. Chan, School of Public Health, Boston, MA, United States of America; Universidad Rey Juan Carlos, SPAIN

## Abstract

Identifying essential proteins is very important for understanding the minimal requirements of cellular life and finding human disease genes as well as potential drug targets. Experimental methods for identifying essential proteins are often costly, time-consuming, and laborious. Many computational methods for such task have been proposed based on the topological properties of protein-protein interaction networks (PINs). However, most of these methods have limited prediction accuracy due to the noisy and incomplete natures of PINs and the fact that protein essentiality may relate to multiple biological factors. In this work, we proposed a new centrality measure, OGN, by integrating orthologous information, gene expressions, and PINs together. OGN determines a protein’s essentiality by capturing its co-clustering and co-expression properties, as well as its conservation in the evolution process. The performance of OGN was tested on the species of *Saccharomyces cerevisiae*. Compared with several published centrality measures, OGN achieves higher prediction accuracy in both working alone and ensemble.

## Introduction

Essential proteins are cellular functional molecules that are indispensable to the survival or reproduction of a living organism. Essential protein identification is crucial for understanding the minimal requirements of basic cell functions, and identifying human disease genes [1] and new drug targets [2]. Experimental methods for the discovery of essential proteins are often time-consuming, laborious, and costly. Computational methods can help to rank the genes based on publicly available biological resources and so greatly reduce the experimental cost needed for finding a novel gene target.

With the accumulation of high-throughput experimental data, it’s now possible to predict protein essentiality in network level. Many researchers have explored the correlations between network topological features and protein essentiality, and found that proteins highly connecting with other proteins in PIN are more likely to be essential than those of low connections. This so-called centrality-lethality rule [3] has been observed in several species, such as *Saccharomyces cerevisiae*, *Caenorhabditis elegans*, and *Drosophila melanogaster* [4–5]. Many centrality measures have been proposed to capture the correlations between network topological properties and protein essentiality, including degree centrality (DC) [5], betweenness centrality (BC) [6], closeness centrality (CC) [7], eigenvector centrality (EC) [8], and subgraph centrality (SC) [9]. Since the existing PINs for many species are not complete and very noisy, the identification of essential proteins solely based on network topology is still very challenging. In addition, protein essentiality is expected to be affected by multiple biological factors, while network topological properties only capture some of its characteristics. Most centrality measures that are only based on PINs could be sensitive to the noise in each PIN, even though they have been found to correlate with the essentiality of proteins.

We need to find out more robust and accurate centrality measures for predicting essential proteins. Recently, several new centrality measures have been proposed by combining topological properties with other biological information. For example, CoEWC [10] and PeC [11] integrated PINs with gene expression data and showed significant performance improvement compared to methods only based on PINs. SON [12] integrated subcellular localization, orthology, and PINs. LBCC [13] integrated local density, betweenness centrality and in-degree centrality of protein complex. GOS [14] integrated gene expression, orthology, subcellular localization and PINs together to predict essential proteins. Besides, Zhang et al proposed an ensemble framework that can significantly improve the prediction accuracy of traditional centrality measures by combining gene expression data and PINs [15]. In general, the integration of network topological properties and additional biological information can improve the prediction accuracy due to the increased robustness by considering multiple biological factors. The advances and challenges in identifying essential proteins using computational methods were reviewed in [16–17].

Essential proteins tend to form highly connected protein clusters rather than function independently [18]. Some recently proposed prediction methods aimed to capture the relationship between essentiality and their cluster property [10–14]. Han et al. found that network hubs in the yeast interactome can be classified into date and party hubs based on their partners’ expression profiles [19]. These two types of hubs are both likely to be essential, although they have very different clustering properties with their neighbors. CoEWC [10] tried to capture the common topological properties of both date and party hubs by focusing on the clustering property of its neighbors rather than the protein itself, and got improved prediction accuracy compared to those for measuring the clustering property of each single protein.

Essential genes tend to be persistent during the long-term evolution [20]. Based on this assumption, Geptop was developed to offer gene essentiality annotations for bacterial organisms using phylogeny weighted orthology information [21]. Some other studies also showed that the integration of orthologous information with topological properties improved the prediction accuracy [12,14].

Having acquired all these recent achievements, we proposed a new centrality measure, OGN, by integrating orthologous information, gene expressions, and PINs together. We implemented OGN to combine the topological properties common to both date hubs and party hubs, the probability of co-expression with the neighboring proteins, and the orthologs in reference organisms. We examined the performance of OGN on data of a well-studied species, *Saccharomyces cerevisiae*. Compared to several previously proposed centrality measures, OGN achieved higher prediction accuracy. Furthermore, we proposed an ensemble method by adjusting the parameter in OGN, which could make OGN usable to other organisms for predicting essential proteins without the trouble of searching optimal parameter for the corresponding organism.

## Methods

In this paper, we use Pearson correlation coefficient (PCC) to capture the co-expression property of a protein with its neighbors, use local clustering coefficient to capture the high connectivity and co-clustering property of a protein, and use orthologous score to capture a protein’s conservation in evolutionary process.

The PPI network is represented by an undirected graph G(*V*, *E*), where a node v∈ *V* represents a protein and an edge *e*(*u*, *v*) denotes an interaction between two proteins *u* and *v*. For a protein *u*, its OGN (*u*) is defined in Eq (1). *PCC*(*u*, *v*) is the Pearson correlation coefficient between two proteins, *u* and *v*, which is calculated based on their gene expression profiles [10]. *Co*(*v*) is the local clustering coefficient of protein *v* which quantifies how close its neighbors are to being a clique (complete graph). The local clustering coefficient of a protein *v* in PPI network is defined in Eq (3), where (*v*, *i*) is the edge weight with definition in Eq (4). *OS*(*u*) is the normalized orthologous score of protein *u*, which is defined as the number of reference organisms which have orthologs of *u* divided by the total number of reference organisms, and is then normalized by dividing the maximal orthologous score across all proteins. *N*_*u*_ is the set of all immediately connected neighbors of node *u* in the PIN, and *kv* denotes the number of neighbors of protein *v*. Parameter α is used to adjust the contributions of the network topological properties of a node (TPN) and its conservation (OS), where 𝛼∈[0, 1].

$$OGN(u)=\alpha \times OS(u)+(1-\alpha )\times \frac{TPN(u)}{max(TPN(x))},\;x\in V$$ (1)

$$TPN(u)=\sum_{v\in N_{u}}PCC(u,v)\times Co(v)$$ (2)

$$Co(v)=\frac{2\sum_{i\in N_{v}}w(v,i)}{k_{v}\times (k_{v}-1)}$$ (3)

$$w(v,i)=\left\{ \begin{array}{cc} 1, & e(v,i)\in E\\ 0, & e(v,i)\notin E \end{array}\right.$$ (4)

From the definition of OGN, we can expect that its performance would be affected by different parameter α. In order to make it easy to apply OGN to different organisms to identify essential proteins and to minimize the selection pressure of parameter α, we also propose a simple ensemble method by utilizing the parameter α. The ensemble method works as follows. For each α_*i*_∈[0, 1], *i* = 1,2, …, *M*, we can get an *OGNi*(*u*) for each protein *u* in the PIN and its corresponding rank. Then we can get *M* ranks for each protein. According to each ranking OGN*i*, we select the top *n* ranked proteins, denoted as X_i_, and combine them as the total candidates set X. We then use ensemble score (ES) and majority voting strategy to further predict essential proteins from X.

For each protein *u* in X, if it’s a member of top *n* ranked proteins based on ranking OGN*i*, that is, *u*∈ X_i_, then its ensemble score *ES*(*u*) increases by 1 (see Eq (5)). I_*i*_(*u*) equals to 1 if *u*∈ X_i_, otherwise 0. In majority voting strategy, the threshold *T* should be equal or larger than half of *M*. According to the ensemble score and the threshold *T*, we further select proteins whose ensemble scores are larger than *T* as the essential candidates of the ensemble method, among which the number of true essential proteins can be determined according to the known protein essentiality. The proposed ensemble method enables us to predict essential proteins for different organisms based on OGN without knowing whether the optimal value for α is same or not for different organisms.

$$ES(u)=\sum_{i=1}^{M}I_{i}(u)$$ (5)

## Results and discussion

### Test data

To evaluate the performance of the proposed OGN centrality measure and the ensemble method, the PIN and gene expression data of *Saccharomyces cerevisiae* were used, as it has been well characterized by knockout experiments and widely used in the evaluation of methods for essential protein discovery. The PPI data was downloaded from BioGRID [22] (version 3.4.143). Gene expression data was retrieved from [23], containing 6,777 gene products and 36 samples. 5,427 proteins were common to the PPI data and gene expression data, which were used for performance evaluation. If a protein/gene had multiple gene expression profiles, the one with maximal mean expression level across the 36 samples was selected. About the selection of gene expression data for predicting essential proteins, we think the following aspects should be considered: 1) sample size; 2) experimental condition; 3) time serials. Generally speaking, larger sample size is preferable because it can more effectively capture gene expression patterns; the experiments that are devoted to specific special treatments would not be suitable since they usually can only get limited number of expressed genes (low coverage); the gene expression profiles are collected from same sample under multiple time points. The collection of gene expression data from [23] spans three cell cycles and has a large coverage of yeast genes, which is suitable for the task of identifying essential proteins.

Essential proteins were collected from several databases, such as SGD [24], DEG [25], and SGDP [26]. 1,194 proteins (S1 Table) are essential among the 5,427 proteins. Orthologous information was collected from InParanoid database (version 7), which contains 100 whole genomes (99 eukaryotes and 1 prokaryote) [27].

### Compare OGN with eight other centrality measures

To validate the performance of OGN, we compared it with several other centrality measures: DC, BC, CC, EC, SC, CoEWC, SON, and LBCC. The five traditional centrality measures (DC, BC, CC, EC, and SC) were used as the baseline since they are solely based on the topological properties of PINs. CoEWC, SON, and LBCC are all utilizing other biological information to improve the prediction accuracy in addition to the network topological characteristics of PINs. We used the reported optimal parameters for SON and LBCC. For SON, we set α = 0.3.

**Fig 1:**
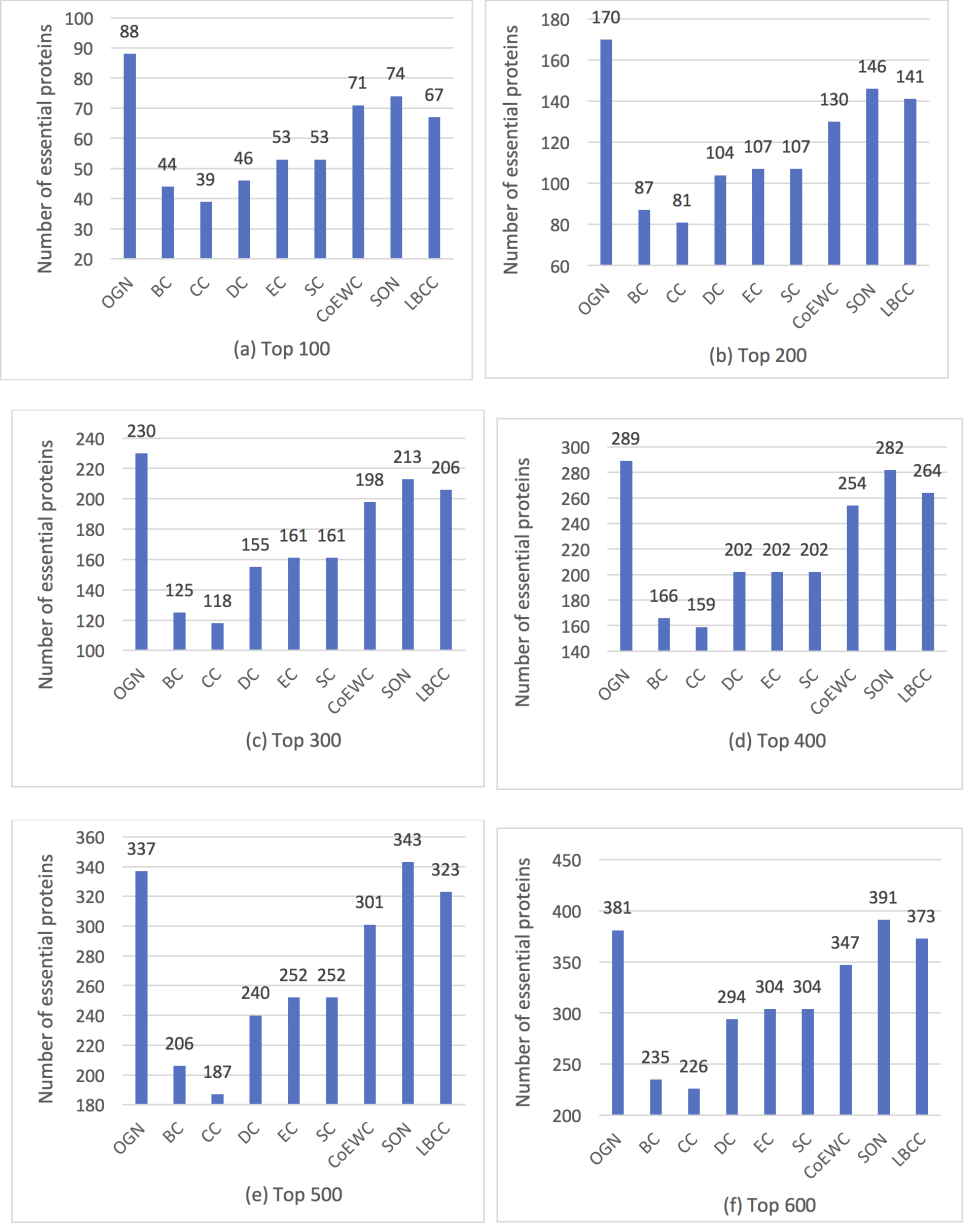
The number of essential proteins predicted by OGN, BC, CC, DC, EC, SC, CoEWC, SON, and LBCC. (a)-(f) show the results of these methods when select top 100 to 600 ranked proteins as candidate essential proteins.

We ranked the proteins in descending order according to each method, and chose the top 100 to top 600 proteins as essential candidates for each method. Then the number of true essential proteins were calculated according to known protein essentiality. The comparison results were shown in Fig 1. We can see that OGN outperforms the other seven methods (DC, BC, CC, EC, SC, CoEWC, and LBCC) significantly. OGN also outperforms SON for top 100 to top 400 predicted essential protein candidates. SON slightly outperforms OGN when considering larger number of predicted candidates. Taking top 100 predicted essential proteins as an example, 88 essential proteins are correctly predicted by OGN, and SON ranks 2^nd^ by correctly predicting 74 essential proteins, while CC performs worst by correctly predicting 39 essential proteins. That is to say, for top 100 predicted essential candidates, OGN obtains about 66% improvements over the 5 traditional centrality measures (BC, CC, DC, EC, and SC), about 24% improvements over CoEWC, about 31.3% improvements over LBCC, and about 19% improvements over SON. For predicting no more than 600 essential candidates, OGN achieves more than 25% improvements compared with the 5 common used centrality measures (BC, CC, DC, EC, and SC), and about 10% improvements over CoEWC.

**Fig 2:**
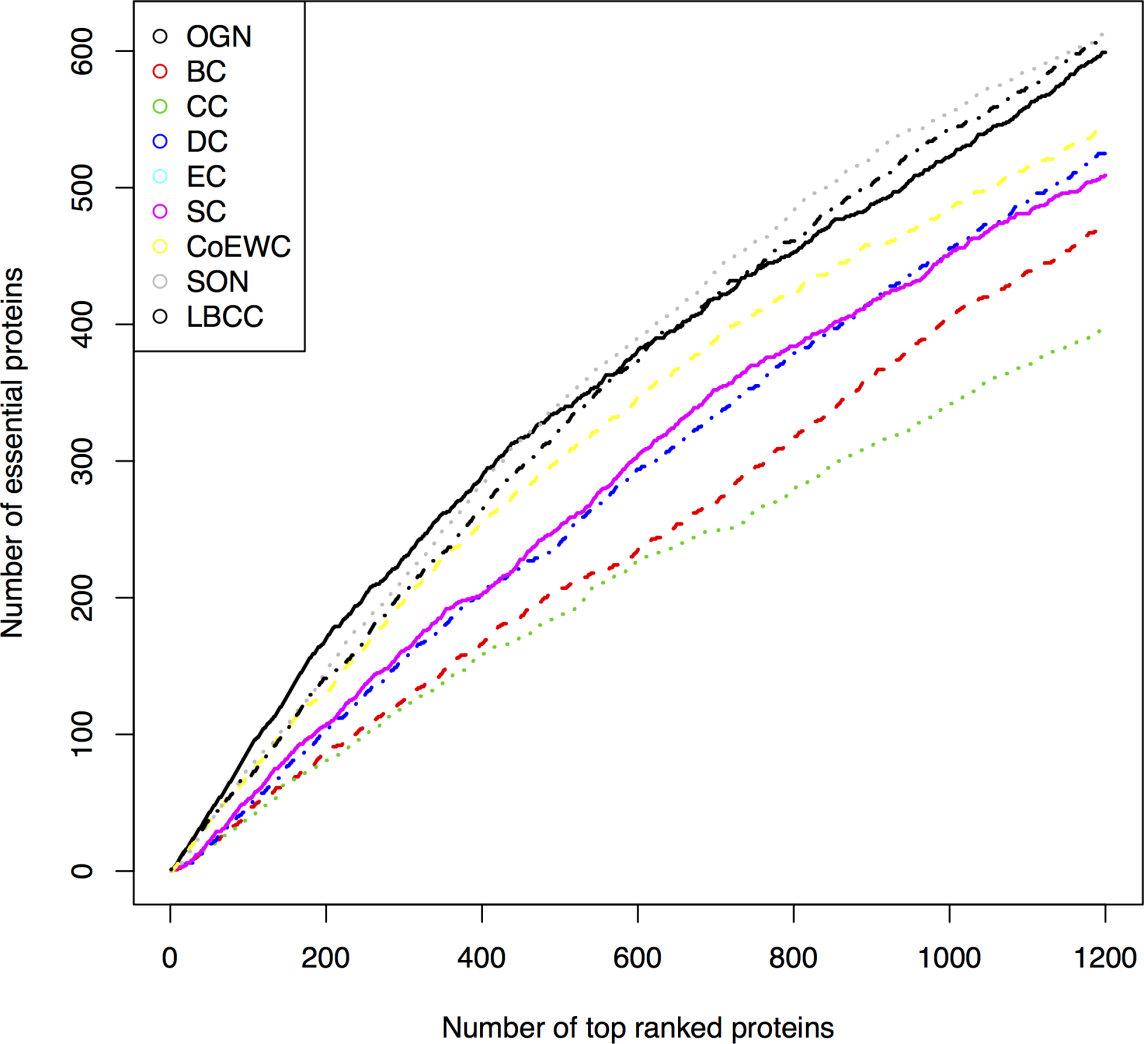
Comparison of OGN, CoEWC, SON, LBCC, and five common used centrality measures (BC, CC, DC, EC, and SC) using Jackknife method.

Fig 2 shows the comparison results of OGN and the other eight compared centrality measures using Jackknife method. In Fig 2, the horizontal axis represents the top *n* ranked essential candidates and the vertical axis represents the accumulation quantity of the correct predictions for each method. From Fig 2 we can see that OGN always performs better than the other six methods (BC, CC, DC, EC, SC, and CoEWC). In addition, OGN outperforms SON when *n* < 450 and outperforms LBCC when *n* < 700. Note that, LBCC is very time consuming which took over 1 day to get the results on our PIN, while OGN only took several minutes. It demonstrates that OGN is effective to predict yeast essential proteins and superior to the other compared centrality measures.

**Fig 3:**
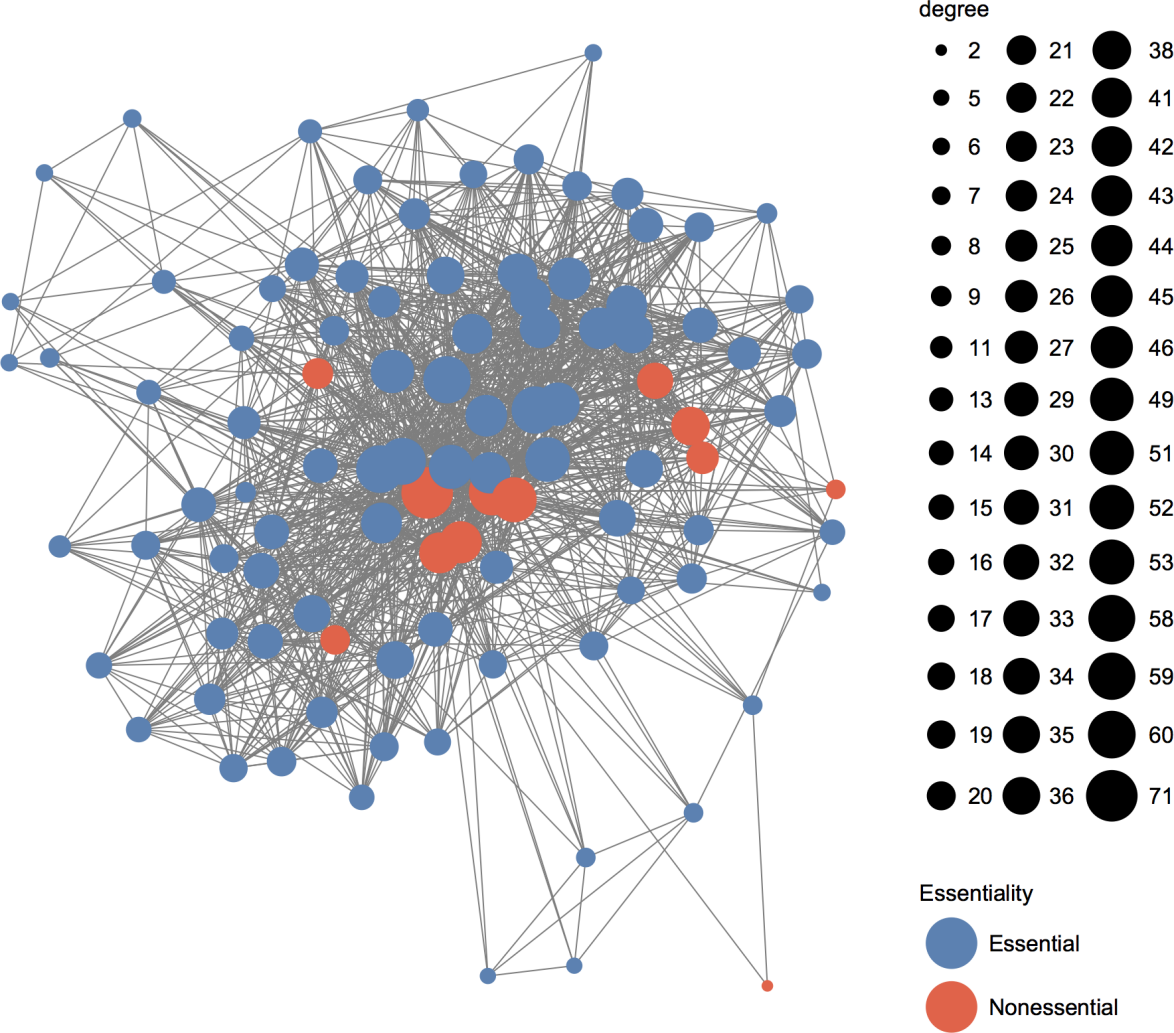
The protein interaction network for the top 100 selected proteins by OGN (alpha = 0.3).

S2 Table shows the top 100 predicted essential candidates by OGN with alpha = 0.3. We also give the corresponding OGN, OS, and DC values as well as the protein essentiality. For the 12 nonessential proteins, they tend to have lager DC values (the degrees range from 53 to 2002 in the PIN) and/or larger OS values, which may in part explain why they are predicted as essential by OGN. Fig 3 shows the subnetwork of the top 100 predicted essential candidates by OGN. From Fig 3 we can see that all the 100 proteins are connected to form one subnetwork, and most of the nonessential proteins have larger degrees which accords with the results shown in S2 Table. In addition, the interaction with multiple essential proteins may play an important role to make these 12 nonessential proteins showing similar characteristics with those of essential proteins. We further examined the 12 nonessential proteins by text mining and database search. YNL255C (GIS2) was confirmed as nonessential gene, but it may have a role in translation regulation under stress conditions [24]. YNL209W (SSB2) is a member of an essential subfamily of hsp70 genes in *S*. *cerevisiae* [28]. YLL013C (PUF3) is a nonessential gene, but the null mutant shows abnormal mitochondrial morphology and movement, in addition, both the null mutation and overexpression confer respiratory growth defects [24]. YKL009W (MRT4) involves in rRNA processing (GO process term); null mutant exhibits slow growth [24]. YER151C (UBP3) is a nonessential gene; null mutants grow slowly, have large cell size, are defective in vacuolar fragmentation, impaired in use of various nitrogen sources [24]. YNR051C (BRE5) is a ubiquitin protease cofactor; null is sensitive to brefeldin A [24]. YDR496C (PUF6) is required at post-transcriptional step for efficient retrotransposition; absence results in decreased Ty1 Gag:GFP protein levels; null causes increased cold sensitivity, decreased nuclear export, protein/peptide accumulation, and transposable element transposition [24]. YGR220C (MRPL9) is component of the large subunit of the mitochondrial ribosomal, which mediates translation in the mitochondrion; null causes absent respiratory growth, decreased competitive fitness [24]. YDR012W (RPL4B, unclear essentiality status) is subunit of the cytosolic large ribosomal subunit; involved in translation. YBL072C (RPS8A) is subunit of the cytosolic small ribosomal subunit; involved in maturation of the subunit rRNA and translation; null causes decreased resistance to chemicals and decreased competitive fitness [24]. YHL004W (MRP4) is component of the small subunit of the mitochondrial ribosome, which mediates translation in the mitochondrion; null causes decreased innate thermotolerance and decreased resistance to chemicals [24]. YPL178W (CBC2) involves in mRNA splicing, via spliceosome; null causes decreased competitive fitness [24]. For the 12 nonessential genes, some of them may be fitness genes.

### Influence of parameter *α* on OGN

**Fig 4:**
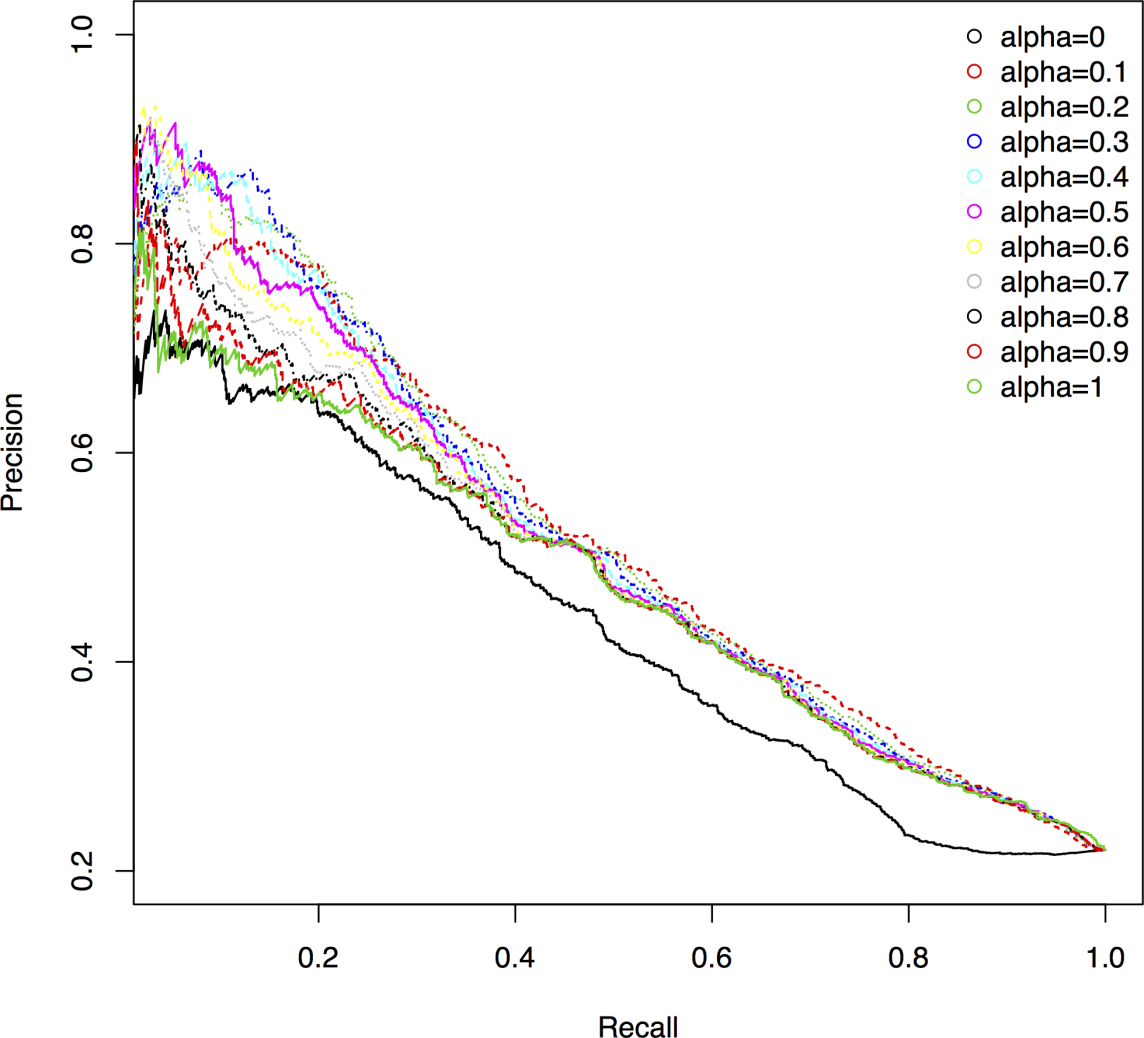
Precision-recall curves of OGN with different α.

From the definition of OGN, we can see that the parameter α adjusts the effect of orthologous information and topological properties. Larger α means that we put more emphasis on orthologous information rather than on topological properties to determine protein essentiality. To analyze the effect of the parameter α on the performance of OGN, we set α∈[0, 1] and observe the number of true essential proteins identified by OGN for top *n* ranked essential candidates. The results are shown in Table 1. We can see that OGN performs worst when α = 0 or 1, which indicates that both the orthologous information and the topological properties contribute to the final results. OGN gets similar performance when α varies from 0.2 to 0.6 while it performs best when α = 0.3. Fig 4 shows the precision-recall curves for OGN with different parameter α. From Fig 4, we can get similar conclusions with those from Table 1.

**Table 1:** The number of true essential proteins identified by OGN with different α.

topn	α = 0	α = 0.1	α = 0.2	α = 0.3	α = 0.4	α = 0.5	α = 0.6	α = 0.7	α = 0.8	α = 0.9	α = 1
100	71	77	86	**88**	87	87	87	82	78	71	70
200	130	159	164	**170**	165	157	152	148	146	141	138
300	198	**234**	232	230	232	227	217	215	205	198	196
400	254	282	**290**	289	284	280	277	271	269	266	257
500	301	**339**	338	337	332	328	326	320	316	314	313
600	347	**385**	382	381	377	376	368	367	363	361	362

### Ensemble performance of OGN with different parameter α

We further evaluate the ensemble performance of OGN with different parameter α. For convenience, we use OGN_*i*_ to indicate the OGN method with parameter α = *i* with *i∈*[0,1]. For α = 0, 0.1,…, 0.9, 1, we get 11 rankings for each protein *u*, OGN_0_[*u*], OGN_1_[*u*],…, OGN_10_[*u*]. Based on each OGN_*i*_, we select the top *n* ranked proteins and combine them as the total candidates set X. According to the ensemble score and the majority voting threshold *T*, a set of proteins whose ensemble scores are larger than *T* are selected as the essential candidates of the ensemble method, among which the number of true essential proteins can be determined according to the known protein essentiality.

**Fig 5:**
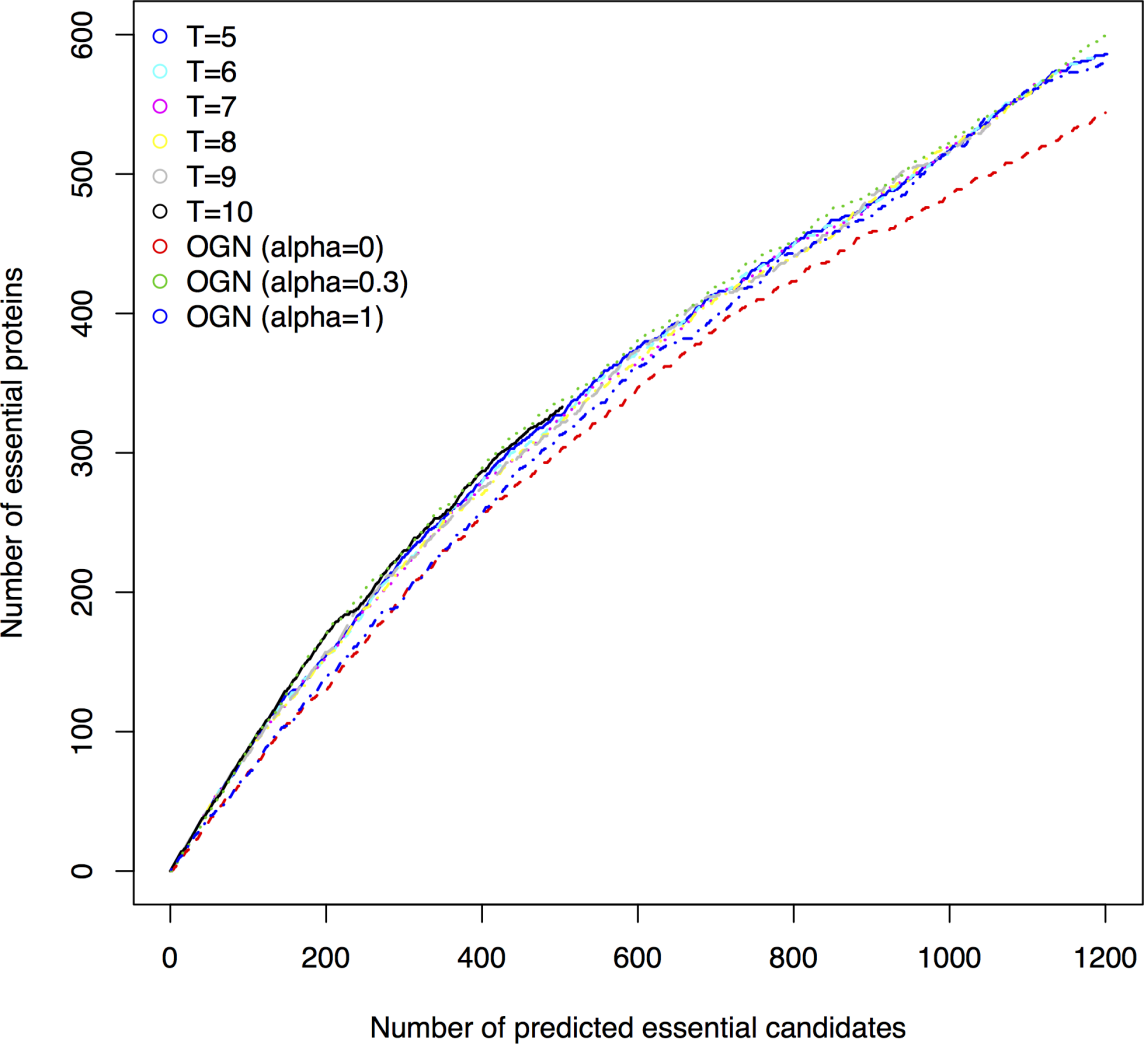
Comparison of the ensemble method with different threshold *T* and OGN (α = 0, 0.3, and 1) using Jackknife method.

Table 2 gives the performance of the ensemble method with different top *n* and thresholds *T*. For example, when *n* = 100 and *T* = 5, 90 proteins are predicted as essential candidates by the ensemble method, among which 78 proteins are true essential, so the precision is 0.867. According to Table 2, the precision increases with the increase of threshold *T* for each *n*, while the number of selected candidates decreases. We further compared the performance of the ensemble method with different threshold *T* using jackknife method. For each ensemble method, its base method OGNs select their top *n* (*n* ranges from 1 to 1200) ranked proteins as the essential candidates, among which the number of predicted essential candidates and the number of true essential proteins predicted by the ensemble method were calculated. Fig 5 shows the performance comparison of the ensemble method with different threshold *T* using Jackknife method. According to Table 1, OGN with α = 0.3 performs best, while OGN with α = 0 or 1 performs worst. We also include the performance of OGN when α = 0, 0.3, and 1 in Fig 5 for comparison convenience. From Fig 5 we can see that the ensemble methods with *T* from 5 to 9 perform similarly; when *T* = 10, it performs best (better precision), but it can only obtain 503 candidates when its base OGN with top *n* = 1200. The ensemble method outperforms OGN with α = 0 and 1. The ensemble method with *T* = 10 performs similarly or slightly better than OGN with α = 0.3 when the number of selected candidates is less than 200.

**Table 2:** Performance of ensemble method with different top *n* and threshold *T*.

Top *n*		*T* = 5	*T* = 6	*T* = 7	*T* = 8	*T* = 9	*T* = 10
100	#predicted	90	69	49	34	15	4
	#true	78	62	45	31	14	4
	Precision	0.867	0.899	0.918	0.912	0.933	1
200	#predicted	192	153	115	92	62	29
	#true	150	125	99	81	55	26
	Precision	0.781	0.8175	0.861	0.88	0.887	0.897
300	#predicted	295	257	222	172	121	65
	#true	222	194	168	133	102	56
	precision	0.753	0.755	0.757	0.773	0.843	0.862
400	#predicted	399	373	330	282	202	116
	#true	279	264	235	207	157	102
	precision	0.699	0.708	0.712	0.734	0.777	0.879
500	#predicted	504	471	436	384	293	167
	#true	328	312	294	266	218	144
	precision	0.651	0.662	0.674	0.693	0.744	0.862

#: the number of. #predicted: the number of predicted essential proteins; #true: the number of true essential proteins.

## Conclusion

In this paper, we proposed a new method for identifying essential proteins, OGN, and tested it on yeast PIN and the related gene expression data as well as orthologs. We compared it with five commonly used centrality measures, BC, CC, DC, SC, and EC, and three integrated methods, CoEWC, SON, and LBCC. The comparison results showed that OGN significantly outperformed these six methods (BC, CC, DC, EC, SC, and CoEWC) for predicting essential proteins. OGN also outperformed SON when *n* < 450 and outperformed LBCC when *n* < 700. In addition, OGN showed similar performance by varying α from 0.2 to 0.6, which indicated that OGN is quite robust to the selection of parameter α.

We also proposed an ensemble method using OGN with different parameter α, which outperformed the best performed OGN (α = 0.3) when the number of selected essential candidates was less than 200, and outperformed the worst performed OGNs with α = 0 or 1. This indicated that the ensemble method is a reasonable alternative when we don’t know the optimal parameter α. Note that, the ensemble method only used the simple majority voting strategy, there would be more performance improvement by integrating multiple features using more sophisticated machine learning methods [16, 29–30].

## Supporting information

S1 Table The essential proteins/genes. (XLSX)

S2 Table The top 100 predicted essential candidates by OGN with (α = 0.3). (XLSX)
